# Severe SARS-CoV-2 Infection in a Cat with Hypertrophic Cardiomyopathy

**DOI:** 10.3390/v13081510

**Published:** 2021-07-31

**Authors:** Francisco R. Carvallo, Mathias Martins, Lok R. Joshi, Leonardo C. Caserta, Patrick K. Mitchell, Thomas Cecere, Sandy Hancock, Erin L. Goodrich, Julia Murphy, Diego G. Diel

**Affiliations:** 1 Department of Biomedical Sciences & Pathobiology, Virginia-Maryland College of Veterinary Medicine, Virginia Tech, Blacksburg, VA 24061, USA; fcarvallo@vt.edu (F.R.C.); tcecere@vt.edu (T.C.); 2 Animal Health Diagnostic Center, Department of Population Medicine and Diagnostic Sciences, College of Veterinary Medicine, Cornell University, 240 Farrier Rd., AHDC A3-114, Ithaca, NY 14853, USA; mm3245@cornell.edu (M.M.); lrj36@cornell.edu (L.R.J.); lcc88@cornell.edu (L.C.C.); pkm57@cornell.edu (P.K.M.); elg25@cornell.edu (E.L.G.); 3 Laboratory for Neurotoxicity Studies, Virginia-Maryland College of Veterinary Medicine, Virginia Tech, Blacksburg, VA 24061, USA; skperkin@vt.edu; 4 Virginia Department of Health, Division of Surveillance and Investigation, Richmond, VA 23218, USA; julia.murphy@vdh.virginia.gov

**Keywords:** COVID-19, SARS-CoV-2, cat, hypertrophic cardiomyopathy, comorbidity

## Abstract

Coronavirus disease 19 (COVID-19), has claimed millions of human lives worldwide since the emergence of the zoonotic severe acute respiratory syndrome coronavirus 2 (SARS-CoV-2) in China in December 2019. Notably, most severe and fatal SARS-CoV-2 infections in humans have been associated with underlying clinical conditions, including diabetes, hypertension and heart diseases. Here, we describe a case of severe SARS-CoV-2 infection in a domestic cat (*Felis catus*) that presented with hypertrophic cardiomyopathy (HCM), a chronic heart condition that has been described as a comorbidity of COVID-19 in humans and that is prevalent in domestic cats. The lung and heart of the affected cat presented clear evidence of SARS-CoV-2 replication, with histological lesions similar to those observed in humans with COVID-19 with high infectious viral loads being recovered from these organs. The study highlights the potential impact of comorbidities on the outcome of SARS-CoV-2 infection in animals and provides important information that may contribute to the development of a feline model with the potential to recapitulate the clinical outcomes of severe COVID-19 in humans.

## 1. Introduction

Coronaviruses are associated with systemic, respiratory and/or enteric infections in humans and in livestock (cattle, horses, pigs and poultry) and companion animals (dogs, cats and ferrets) [1]. The members of the *Coronaviridae* are recognized for their ability to cross the species barrier and establish new host reservoirs of infection [2]. The pandemic severe acute respiratory syndrome coronavirus 2 (SARS-CoV-2), which causes coronavirus disease 19 (COVID-19) in humans, has likely spilled over from bats to a yet unknown intermediate animal species and then to humans [3,4]. Interestingly, natural SARS-CoV-2 infections have been reported in dogs, cats, tigers, lions, snow leopards, a cougar, puma, gorillas, ferrets and farmed and wild caught mink [5,6,7,8,9,10,11]. All animal cases have been shown to have an epidemiological link to confirmed human cases of COVID-19, suggesting human-to-animal transmission. Importantly, infections with SARS-CoV-2 in most animals have been associated with subclinical or mild respiratory distress. 

In humans, most severe COVID-19 cases and deaths have been associated with underlying medical conditions, including cancer, kidney disease, chronic respiratory diseases, obesity, diabetes and heart disease (such as heart failure, coronary artery disease or cardiomyopathies) [12]. The association of underlying cardiovascular disease (CVD) and myocardial injury with severe and often fatal outcomes in COVID-19 patients has been well documented in humans [13,14]. However, the physio pathological mechanisms underlying these conditions and their contribution to the pathogenesis of severe COVID-19 are still under investigation.

Here, we describe a case of severe respiratory and myocardial SARS-CoV-2 infection in a domestic cat (*Felis catus*), which suffered from hypertrophic cardiomyopathy (HCM), a common comorbidity of COVID-19 [15,16]. Virological and pathological findings demonstrated viral replication and virus associated lesions in multiple tissues and organs including lung and heart, an outcome that resembles multi-systemic involvement in severe cases of COVID-19 in humans. 

## 2. Materials and Methods

### 2.1. Case History

A four-year-old male, neutered domestic medium-hair cat, became ill on December 7 2020 after a presumed exposure to a person living in the same household who became ill on December 4 and was antigen test positive for SARS-CoV-2 on December 5. The owner reported that on December 7, the cat’s appetite decreased which was followed by lethargy beginning on December 11 and respiratory distress beginning on December 13. The second household member also tested positive for SARS-CoV-2 by rRT-PCR. Due to the progressive and severe respiratory disease presented by the cat, the owner elected for humane euthanasia of the animal on December 15. Two other pets (a cat and a dog) in the same household did not develop any clinical signs. No further testing was performed in these animals. 

### 2.2. Diagnostic Investigation

A full necropsy was performed on the animal. Following euthanasia, the body was immediately refrigerated, and the necropsy was performed 72 h after euthanasia. A fragment of right cranial lung lobe was submitted to microbiology for aerobic culture. Swabs from the nasal cavity, oropharynx, trachea and rectum were collected in phosphate buffer-saline solution, as well as fresh samples of lung, heart, liver, spleen, kidney and small intestine were frozen and submitted to the Animal Health Diagnostic Center at Cornell University for real-time reverse transcriptase PCR (rRT-PCR) testing for SARS-CoV-2 and PCR and virus isolation for other common feline respiratory pathogens. Samples of nasal turbinate, trachea, thyroid gland, lung, heart, diaphragm, liver, spleen, pancreas, adrenal gland, kidney, urinary bladder, esophagus, stomach, small intestine, mesenteric lymph node, cecum, colon and brain were collected and processed for histological evaluation. All tissues were fixed in 10% neutral buffered formalin, pH 7.2, for 48 h and processed by routine histological methods. Tissues were sectioned (5 µm thick), stained with hematoxylin and eosin and subjected to histological evaluation. Sections of the lung were also analyzed with PTAH stain.

### 2.3. SARS-CoV-2 and Feline Respiratory Pathogen PCRs

Total nucleic acid was extracted from clinical samples (respiratory secretions and tissues) using the the MagMax Core extraction kit (Thermo Fisher, Waltham, MA, USA) and the automated KingFisher Flex nucleic acid extractor (Thermo Fisher) as previously described [17]. The rRT-PCRs for SARS-CoV-2 were performed using the EZ-SARS-CoV-2 real-time PCR assay following the manufacturer’s instructions (Tetracore Inc., Rockville, MD, USA). This assay detects both genomic and subgenomic viral RNA for increased diagnostic sensitivity. An internal inhibition control was included in all reactions and positive and negative amplification controls were run with test samples. All RNA extractions and rRT-PCRs were performed at the Cornell Animal Health Diagnostic Center (AHDC).

The respiratory swab specimens were also subjected to diagnostic testing for other common feline respiratory pathogens, including *Bordetella* sp., *Chlamydia felis*, *Mycoplasma cynos*, *Mycoplasma felis*, *Streptococcus equi*. ssp. *zooepidemicus*, Influenza virus, pneumovirus, feline calicivirus and feline herpesvirus at the Cornell AHDC. 

### 2.4. Virus Isolation and Titrations

The samples that tested positive for SARS-CoV-2 by rRT-PCR, including nasal-, oropharyngeal-, tracheal- and rectal swabs and tissues (lung, heart, kidney liver, spleen and small intestine) were subjected to virus isolation under biosafety level 3 conditions at the Cornell AHDC. For this, twenty-four well plates were seeded with ~75,000 Vero-E6/TMPRSS2 cells per well 24 h prior to sample inoculation. Cells were rinsed with phosphate buffered saline (PBS) (Corning^®^) and inoculated with 150 µL of each sample (swab supernatant or tissue homogenate [10% weight/volume]) and the inoculum adsorbed for 1 h at 37 °C with 5% CO_2_. Mock-inoculated cells were used as negative controls. After adsorption, cells were rinsed with PBS and replacement cell culture media (Dulbecco’s modified eagle medium (DMEM), supplemented with 10% fetal bovine serum (FBS), L-glutamine (2 mM), penicillin (100 U·mL^−1^), streptomycin (100 μg·mL^−1^) and gentamicin (50 μg·mL^−1^)) was added and cells were incubated at 37 °C at 5% CO_2_ and monitored daily for viral cytopathic effect (CPE) for 3 days. Cell cultures with no CPE were frozen, thawed and subjected to two additional blind passages/inoculations in Vero-E6/TMPRSS2 cell cultures. Cell cultures presenting viral CPE and those negative at the end of the third passage were subjected to an immunofluorescence assay (IFA) as follows. At 24 h post-inoculation, cells were fixed with 3.7% formaldehyde for 30 min at room temperature, permeabilized with 0.1% Triton X-100 (in PBS) and subjected to IFA using a rabbit polyclonal antibody specific for SARS-CoV-2 nucleoprotein (N) and a mouse monoclonal antibody specific for the S2 subunit of the spike protein (clone DL153-19). Cells were incubated with the N- and S2-specific antibodies for 1 h at room temperature (rt), followed by 1 h incubation at rt with a goat anti-rabbit IgG (goat anti-rabbit IgG, Alexa Fluor^®^ 488) and a goat anti-mouse IgA+IgG+IgM secondary antibody (goat anti-mouse IgG, Alexa Fluor^®^ 594). Next, nuclear counterstain was performed with DAPI and cells visualized under a fluorescence microscope.

Virus isolation positive samples were subjected to end point titrations by limiting dilution using Vero-E6/TMPRSS2 cells. For this, the original sample or tissue homogenate (10% *w*/*v* solution) was serially diluted in DMEM (10-fold dilutions) and individual dilutions were inoculated into Vero-E6/TMPRSS2 culture in 96-well plates. At 48 h post-inoculation, cells were fixed and subjected to IFA using the SARS-CoV-2 N-specific polyclonal antibody as described above. Virus titers were determined based on fluorescence positive wells using the Spearman and Karber’s method and expressed as TCID50.mL^−1^.

### 2.5. In Situ Hybridization

Formalin-fixed paraffin-embedded (FFPE) tissues were used to detect SARS-CoV-2 RNA using a 20-oligonucleotide probe targeting the S gene of Wuhan Hu-1 (NC_045512.2; Advanced Cell Diagnostics catalog no. 848561). A probe targeting feline host protein peptidylprolyl isomerase B (PPIB) was used as a positive control (Advanced Cell Diagnostics catalog no. 455011). A probe targeting DapB gene from Bacillus subtilis strain SMY was used as a negative control (Advanced Cell Diagnostics catalog no. 310043) (Figure S1A). The in-situ hybridization assay was performed using RNAscope 2.5 HD Detection Kit Red according to the manufacturer’s instructions (Advanced Cell Diagnostics catalog no. 320751).

### 2.6. Immunohistochemistry

Immunohistochemical staining for SARS-CoV-2 was performed using Vectastain Elite ABC Peroxidase (HRP) Kit (Vector Laboratories catalog no. PK-6102). Formalin-fixed paraffin-embedded (FFPE) tissues were deparaffinized with xylene and rehydrated through a series of graded alcohol solutions. Antigen unmasking was performed using Tris-based antigen unmasking solution (pH 9.0) by boiling the slides in the unmasking solution for 15 min (Vector Laboratories, catalog no. H-3301). Quenching of endogenous peroxidase was performed using 0.3% hydrogen peroxide solution (Abcam catalog no. ab64218). A mouse monoclonal antibody targeting nucleoprotein (N) of SARS-CoV-2 was used as a primary antibody (SARS-CoV-2 NP mAb clone B6G11). For SARS-CoV-2 detection, tissue sections were incubated with anti-mouse biotinylated secondary antibody followed by incubation with the Vectastain Elite ABC HRP reagent. Finally, tissues sections were incubated with Vector DAB peroxidase substrate (3,3′ –diaminobenzidine) with nickel to produce grey-black reaction product (Vector Laboratories catalog no. SK-4100). Counterstaining was performed using Hematoxylin QS Counter stain solution (Vector Laboratories catalog no. H-3404). Tissues from the uninfected cat were used negative controls (Figure S1B).

To characterize the inflammatory infiltrate of the lung and trachea, immunohistochemistry for Pancytokeratin (Roche diagnostics, ready to use), Iba-1 (Rabbit, 1:1000, Wako, Osaka, Japan), CD3 (Rabbit, 1:100, Dako, Trappes, France) and CD79a (Mouse, 1:50, Santa Cruz) was performed following Standard operating procedures at the Virginia Tech Animal Laboratory Services (ViTALS), Virginia Maryland College of Veterinary Medicine using a BenchMark Ultra IHC/ISH system (Roche, Basel, Switzerland). All reactions were detected with Ultraview red detection kit (Roche Diagnostics, Indianapolis, IN, USA) and counterstained with Mayer’s hematoxylin following the manufacturer instructions.

### 2.7. Transmission Electron Microscopy

Heart samples were collected from the left ventricle and fixed in 10% neutral buffered formalin. For electron microscopy, samples were trimmed into 1 mm cubes with a secondary fixation in 3% glutaraldehyde in phosphate buffer, followed by post-fixation in 2% osmium tetroxide, dehydration in a graded ethanol series and embedded in epoxy resin. Ultrathin sections (70–80 nm) were stained with alcoholic uranyl acetate and lead citrate and examined with a JEOL JEM-1400 transmission electron microscope at an accelerating voltage of 80 kV. Digital images were obtained using a Gatan Orius SC1000 Model 832 CCD digital camera with Gatan Microscopy Suite Digital Micrograph software. 

### 2.8. Whole Genome Sequencing and Phylogenetic Analysis

Whole genome sequencing (WGS) of SARS-CoV-2 was performed directly on respiratory secretions and on feces from the affected cat as previously described [7]. For this nucleic acid extracted from the nasal, tracheal, oropharyngeal and rectal swabs from the animal was subjected to MinION-based targeted SARS-CoV-2 WGS. A multiplex RT-PCR was developed following the amplicon-based approach used by the ARTIC Network for sequencing SARS-CoV-2 (https://artic.network/ncov-2019 (accessed on 3 March 2020)). Forty custom primers were designed using Primer3 [18] using the Geneious Prime 2019 software (https://www.geneious.com (accessed on 3 March 2020)). Each primer set amplifies approximately 1500bp long products with 100bp overlaping regions and covering the entire SARS-CoV-2 genome. First-strand cDNA synthesis was performed with the Super Script IV First-Strand Synthesis System (ThermoFisher) using 11 μL of RNA and 1 μL of random hexamer primers at 50 μM. An initial denaturation step was carried out at 65 °C for 5 min and samples were placed on ice for at least 1 min. After adding the cDNA synthesis mix, the mixture was incubated at 23 °C for 10 min, 50 °C for 10 min, 55 °C for 10 min and 80 °C for 10 min. Then, 2.5 µL cDNA was added in 2 separate PCR reactions performed with the Q5 High-Fidelity 2X Master Mix (New England Biolabs). The PCR amplification conditions used were as follows: 30 s at 98 °C, then 40 cycles of 94 °C for 10 s, 60 °C for 1:30 min and 72 °C for 3 min, with a final extension at 72 °C for 2 min. Libraries were generated using the Native Barcode Kit, EXP-NBD104, Ligation Sequencing Kit, SQK-SQK109 (Oxford Nanopore Technologies, Oxford, UK) and sequenced on a R9.4 flow cell for 6 h. 

Raw reads were basecalled and demultiplexed with the MinIT device (Oxford Nanopore Technologies, Oxford, UK). NanoFilt [19] was used for filtering reads by quality and size, and to remove primer sequences from the ends of reads. Reads were assembled using the ARTIC nCoV-2019 Nanopore bioinformatics pipeline (https://artic.network/ncov-2019/ncov2019-bioinformatics-sop.html (accessed on 7 January 2021)) with Medaka (https://github.com/nanoporetech/medaka (accessed on 7 January 2021)) variant calling. Samples with <27,000 bases called were excluded from further analysis. Consensus sequences, along with the Wuhan-Hu-1 reference genome (NC_045512.2) were aligned using mafft v. 7.453 and a phylogenetic tree was built using the GTR-Gamma model in RAxML v. 8.2.9. VCF files were manually inspected at variable positions in the alignment to assess the presence of minor variants.

## 3. Results

### 3.1. Clinical Description and Pathological Observations

In December 2020, a 4.5-year-old, 4.7 kg male castrated domestic medium hair cat presented to a veterinary clinic with a five-day long history of hyporexia and severe progressive respiratory distress that was not responsive to antibiotic treatment. Blood work revealed azotemia and hyperglycemia. Prior to this episode the cat had been diagnosed with a systolic II/IV (faint but easily audible) heart murmur but received no medications. Following the respiratory episode with poor prognosis and progressive severity, the owner elected to perform humane euthanasia of the animal. The animal was submitted to ViTALS, Virginia Maryland College of Veterinary Medicine for diagnostic evaluation. The cat came from a household in which one person tested positive for SARS-CoV-2 by a rapid antigen-capture test ~7 days prior to the initiation of the clinical signs in the cat, while a second person in the household tested positive by real-time PCR ~3–4 days after the cat became ill. 

**Figure 1:**
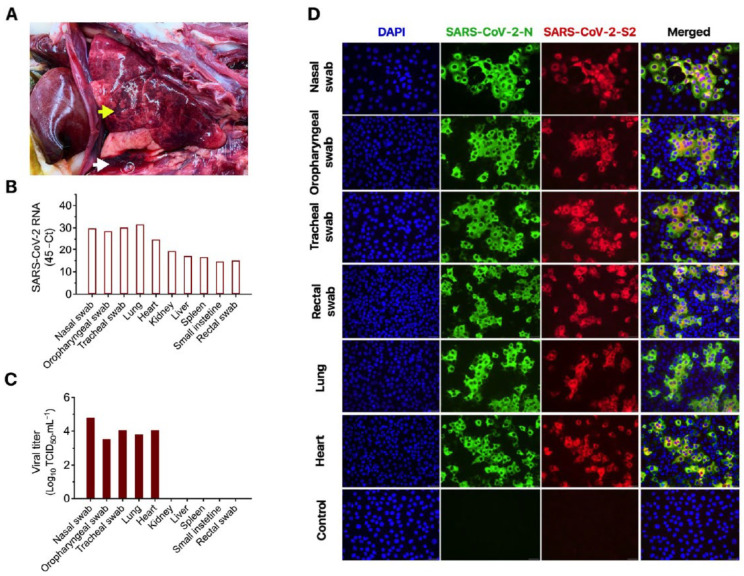
Gross pathological and virological findings in a domestic cat presenting progressive respiratory disease. (**A**) Lungs are mottled red with focal depressed areas in the parenchyma (yellow arrow). A red tinged fluid is present in the thoracic cavity (white arrow). (**B**) Viral RNA detected in respiratory secretions, feces and in lung, heart, kidney, spleen and intestine as determined by real-time RT-PCR. (**C**) Viral titers in respiratory secretions, in lung and heart as determined using end-point titrations and the Spearman and Karber’s method and expressed as TCID_50_.mL^−1^. (**D**) Virus isolation was confirmed by immunofluorescence. For this, Vero-E6/TMPRSS2 cells were inoculated with cell lysate of virus isolation assays performed in respiratory secretions, feces, lung and heart tissue homogenates. At 24 h post-inoculation cells were fixed and subjected to IFA using a polyclonal antibody anti-SARS-CoV-2-nucleoprotein (N) (green), a mAb anti-SARS-CoV-2-Spike protein S2 subunit (red) and the nuclear counterstain performed with DAPI (blue). This confirmed isolation of SARS-CoV-2 from nasal secretions, oropharyngeal and tracheal secretions, feces and from the lungs and heart of the cat. 40× magnification.

At necropsy, a small amount of red tinged fluid was observed in the trachea. Approximately 15 mL of red tinged fluid was collected from the thorax. The lungs were diffusely mottled red and slightly firm, with patchy areas of depression of the parenchyma (Figure 1A). Importantly, the left ventricular wall was moderately thickened, with a narrow ventricular cavity with the heart weighing approximately 22 g. These observations suggested that the animal suffered from HCM, which was confirmed based on histological findings. As mentioned above, prior to the respiratory episode that led to euthanasia of the cat, a systolic II/IV (faint but easily audible) heart murmur had been observed in the animal; however it was not receiving any medication. The liver was slightly pale with a prominent reticular pattern and the left kidney had a focal irregular chronic infarct of the cranial pole (1 cm diameter). No significant gross changes were observed in other organs.

### 3.2. Molecular, Bacteriological and Virological Diagnostic Investigation

Given the clinical and pathological presentation, a complete diagnostic investigation, including viral and bacterial testing and histopathological evaluation, were performed. Respiratory specimens (nasal, tracheal and oropharyngeal swabs) and tissues were submitted to the Cornell AHDC and initially subjected to PCR testing or culture for common feline respiratory pathogens (*Bordetella sp*., *Chlamydia felis*, *Mycoplasma cynos*, *Mycoplasma felis*, *Streptococcus equi.* ssp *zooepidemicus*, Influenza virus, pneumovirus, feline calicivirus and feline herpesvirus). The nasal swab sample tested positive for *M. felis* (Ct = 32), while no other common feline pathogen was detected nor isolated. Additionally, bacterial culture on the lung tissue did not result in bacterial growth. Formalin-fixed paraffin embedded heart and lung tissues were also tested by RT-PCR for feline coronavirus (FeCoV), the causative agent of feline infectious peritonitis, and the virus was not detected in these tissues. Importantly, nasal, oropharyngeal and rectal swabs were positive for SARS-CoV-2 when tested by rRT-PCR (Figure 1B). These results were confirmed at the National Veterinary Services Laboratory (NVSL). Tissue samples including lung, heart, kidney, liver, spleen and small intestine also tested positive for SARS-CoV-2 RNA by rRT-PCR (Figure 1B). Higher viral loads, as evidenced by lower cycle threshold values, were detected in nasal, tracheal and oropharyngeal swabs and in the lung and heart (Figure 1B). 

Virus isolation and titrations performed in SARS-CoV-2 rRT-PCR positive samples revealed shedding of high amounts of infectious virus in respiratory secretions (titers between 3.5 and 4.88 log10 TCID_50_.mL^−1^) (Figure 1C). Infectious virus was also detected in rectal swab samples (Figure 1D). Virus isolation was confirmed using a SARS-CoV-2 specific polyclonal antibody against the N- and a mAb against S2 proteins (Figure 1D). Additionally, high infectious viral loads were detected in the lung and heart of the animal (Figure 1B), suggesting active virus replication in these organs.

### 3.3. Characteristic COVID-19 Lesions Observed in Lung and Heart of the Affected Cat

**Figure 2:**
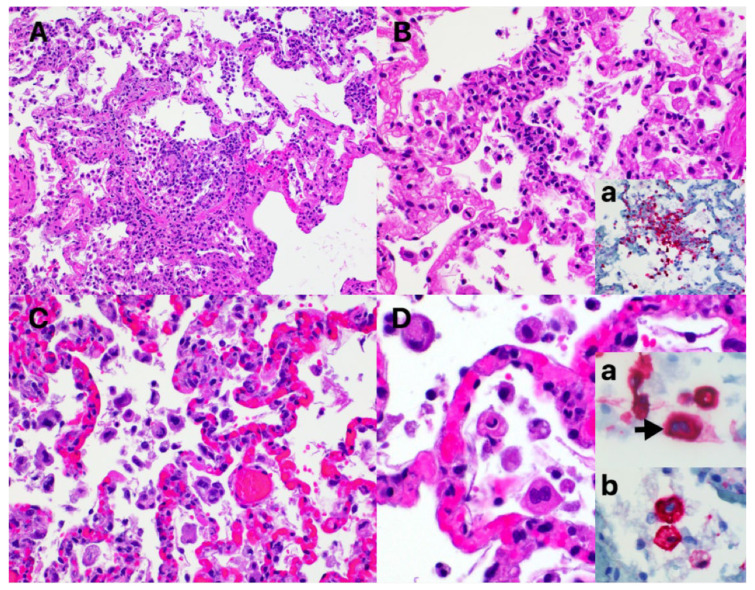
Histological changes in the lung of the SARS-CoV-2 infected cat. (**A**) Necrotizing bronchiolitis and diffuse alveolar damage (DAD), with a partially occluded bronchiole with necrotic cellular debris and inflammatory cells. Adjacent alveolar spaces contain increased cellularity, mild edema and fibrin strands and the interstitium is expanded with numerus inflammatory cells. Hematoxylin and eosin. 20× magnification. (**B**) Capilaritis. Alveolar septa are infiltrated with neutrophils and macrophages. A fibrin thrombus is identified adjacent to the area of inflammation (arrow). Hematoxylin and eosin. Inset a. Iba-1 immunohistochemistry of an area of capillaritis, highlighting the presence of numerous macrophages. Mayer’s hematoxylin counterstain. 40× magnification. (**C**) Alveolar spaces containing increased numbers of mononuclear cells and necrotic cellular debris. The alveolar septa are markedly congested with increased numbers of neutrophils and macrophages. Hematoxylin and eosin. 20× magnification. (**D**) Reactive atypia. Cells present in alveolar spaces, with abundant eosinophilic cytoplasm and moderately enlarged nucleus. Hematoxylin and eosin. 40× magnification. Inset a. Pancytokeratin immunohistochemistry of alveolar cells, indicative of epithelial cells. One binucleated cell is noted (syncytium, arrow). Mayer’s hematoxylin counterstain. Inset b. Iba-1 immunohistochemistry of alveolar cells, indicative of macrophages. Mayer’s hematoxylin counterstain. 40× magnification.

**Figure 3:**
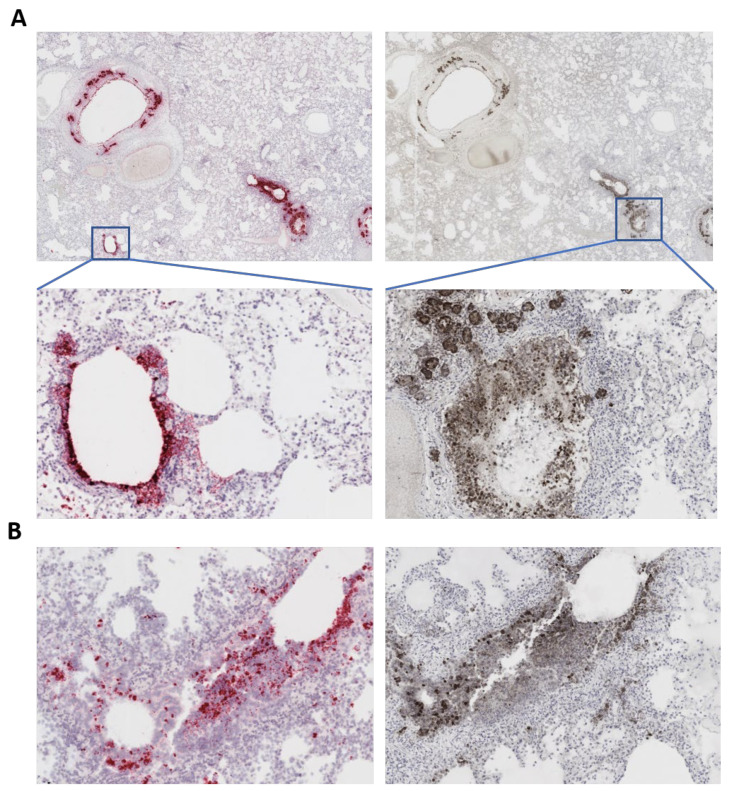
SARS-CoV-2 labeling in the lung of the affected cat. (**A**) In situ hybridization (ISH) (left panels) showing labeling (pink/reddish labeling) for SARS-CoV-2 RNA in bronchial and bronchiolar epithelial cells and bronchial glands. Immunohistochemistry (IHC) (right panels) showing staining for the SARS-CoV-2 N protein (brown stain) in bronchial and bronchiolar epithelial cells and bronchial glands. Tissue section were counter stained with hematoxylin. (**B**) ISH and IHC performed in the same section demonstrating co-localization of viral RNA labeling (pink/reddish) and viral protein (N), indicating active SARS-CoV-2 replication in the lung of the cat.

Tissues were also subjected to complete histopathological evaluations at the ViTALS. Histological lesions consistent with inflammation were found only in the respiratory tract and heart. Examination of the trachea revealed focal areas of tracheal gland necrosis, with infiltrates of neutrophils and macrophages. In the lung, an acute bronchointerstitial pneumonia was noted (Figure 2A,B). In the bronchi, there was circumferential degeneration and necrosis of bronchial epithelial cells, with focal infiltrates of non-degenerate and degenerate neutrophils and macrophages. Similar inflammatory infiltrates were observed surrounding the bronchial glands. Necrosis of epithelial cells and inflammation was identified in the bronchiolar lumen, with some being completely occluded with cellular debris (Figure 2A). In alveoli, diffuse alveolar damage with vascular injury was noted, characterized by numerous macrophages with frequent phagocytosis of cellular debris, presence of multinucleated cells suggesting syncytial cells and a few neutrophils (Figure 2C,D), all immersed in edema, multifocal hemorrhages and fibrin, with the formation of rare hyaline membranes. There was multifocal segmental hyperplasia of type II pneumocytes. The alveolar septa were thickened with increased numbers of intravascular neutrophils and multiple segments of necrotizing capillaritis, with microthrombi and focal dense infiltrates of neutrophils and macrophages (Figure 2B). Occasional arteries had transmural infiltrates of mononuclear cells with margination of neutrophils. Most of the cells present in alveoli, particularly multinucleated cells, presented strong cytoplasmic immunoreactivity for Pan Cytokeratin (Figure 2D, inset a). Most inflammatory cells infiltrating the tracheal glands and approximately one third of alveolar cells presented a strong cytoplasmic immunoreactivity for Iba-1, indicative of macrophages (Figure 2B, inset a and Figure 2D, inset b). Multifocal cluster of CD3 positive cells, indicative of T lymphocytes, were identified surrounding tracheal glands, bronchial glands, bronchioles and blood vessels (data not shown). No B lymphocytes were highlighted with CD79a stain in the analyzed sections of lung or trachea. Importantly, in situ hybridization (ISH) for SARS-CoV-2 RNA and immunohistochemistry (IHC) staining revealed strong viral labeling/immunoreactivity in bronchial epithelial cells, bronchiolar glands, bronchiolar epithelium and in few desquamated epithelial cells/macrophages in alveolar spaces (Figure 3).

**Figure 4:**
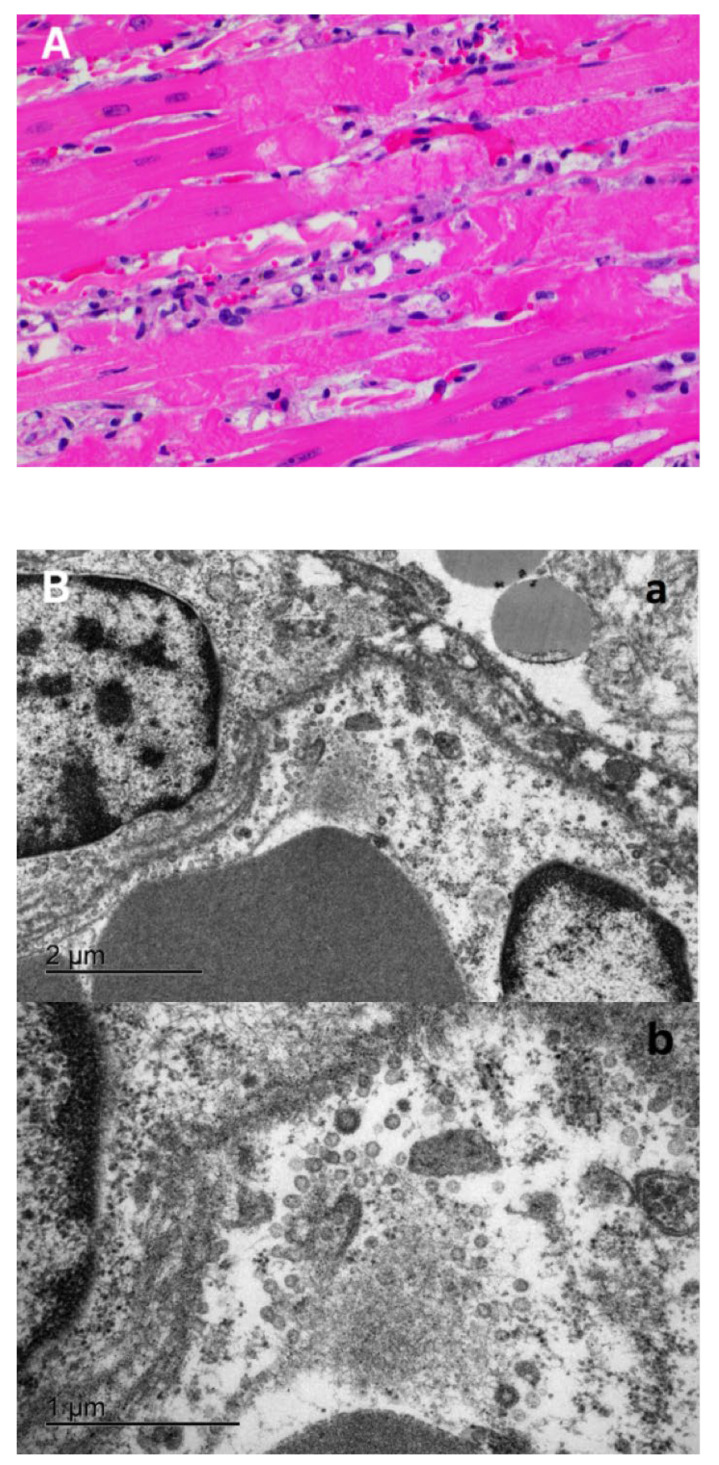
Morphologic changes and viral localization in the heart of the SARS-CoV-2 infected cat. (**A**) Myocardial degeneration and necrosis. Cardiomyocytes with hypereosinophilic sarcoplasm with hypercontraction bands and loss of striations. Small numbers of neutrophils and macrophages in the myocardial interstitium. Hematoxylin and eosin. 40× magnification. (**B**) Electron microscopy of the ventricular myocardium. A blood vessel containing portions of two erythrocytes (lower left) and lined by two endothelial cells, each with a partial profile of the nucleus. Multiple coronavirus-like particles are within endoplasmic reticulum cisternae or are adjacent to free ribosomes and fragmented cytosolic debris (autolysis) (panel a). Bar = 2 µm. Panel b—Higher magnification of panel a. Virus particles are round with an outer double membrane and a core that contains variably distinct electron dense dots representing cross sections of the nucleocapsid. Bar = 1 µm.

In the heart, there were multifocal to coalescing areas of degeneration and necrosis of cardiomyocytes, with cells displaying hypereosinophilic cytoplasm, loss of striations, hyper contraction bands, vacuolation and/or pyknotic nuclear debris (Figure 4A). There was edema with the presence of small numbers of macrophages and neutrophils between myofibers (Figure 4A). In addition, there was mild concentric hypertrophy of the left ventricle. Cardiomyocytes have moderate variation of the cytoplasmic diameter, a feature that was more frequent towards the periphery of the myocardium. In addition to isolation of infectious SARS-CoV-2 from the heart (Figure 1C,D), electron microscopy (EM) examination of the heart tissue revealed multiple coronavirus-like particles in endothelial cells within the ventricular myocardium. The viral particles were evident within cisternae of the endoplasmic reticulum, in membrane bound vesicles adjacent to the inner surface of the plasma membrane, or adjacent to ribosomes and cytosolic debris in cells with autolytic change. Virus particles were composed of an outer double membrane and a core consisting of multiple, variably distinct, electron dense punctate dots (nucleocapsid) (Figure 4B).

Histological evaluation of the mesenteric lymph node, revealed abundant fibrin deposition, multifocal hemorrhages and small numbers of foamy macrophages within the subcapsular sinuses. There was marked congestion of the cortex, with edema and occasional necrotic cellular debris in lymphoid germinal centers (not shown). No significant findings were identified in other internal organs examined.

### 3.4. Comparative Genomic Analysis

The complete genome sequence of SARS-CoV-2 was obtained directly from the respiratory secretions (nasal, tracheal and oropharyngeal secretions) and feces from the affected cat. When compared to the Wuhan-Hu-1, the SARS-CoV-2 virus recovered from the cat presented 14 single nucleotide polymorphisms (SNPs). Lineage prediction using pangolin https://github.com/cov-lineages/pangolin/blob/master/github.com/cov-lineages/pangolin, accessed on 5 March 2021 [20,21,22,23,24,25] identified the sequence as belonging to SARS-CoV-2 lineage B.1.240 which comprises a total of 2033 sequences in the GISAID dataset (accessed on 25 March 2021).

**Figure 5:**
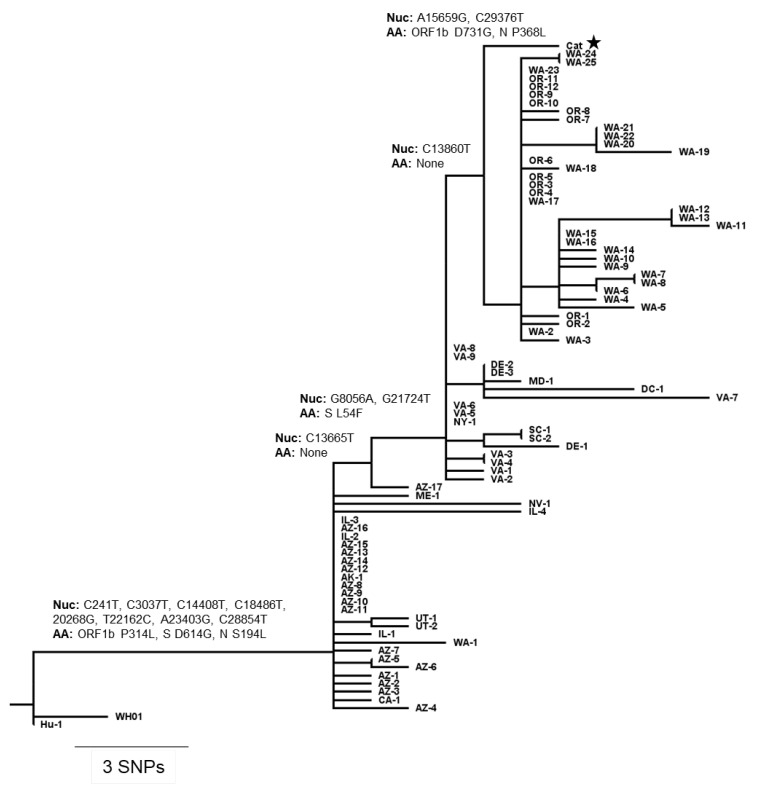
Phylogenetic placement of SARS-CoV-2 obtained from the infected cat. Phylogenetic tree with branches scaled by genetic divergence, with SNPs and amino acid changes labeled on branches leading to the cat sample. Additional sequences, labeled by their state of origin were selected by genetic proximity to the cat sequence from the pool of sequences matching the pangolin B.1.240 lineage. Wuhan/Hu-1/2019 and Wuhan/WH01/2019 were included for rooting. Full names of the included sequences are given in Tables S1 and S2.

The SARS-CoV-2 sequence identified in the cat clustered with human SARS-CoV-2 sequences detected in Virginia or in nearby states. Phylogenetic analysis using Nextstrain [26] placed two SNPs, both encoding amino acid changes (A15659G, ORF1b: D731G; C29376T, N: P368L) on the terminal branch leading to the sequence from the cat. One SNP downstream of the parent node of the cat sequence was a group of 36 sequences from the Pacific Northwest (Oregon and Washington) while moving 1 SNP upstream to the next ancestor node added 17 sequences including 10 from Virginia (Figure 5). Unfortunately, no SARS-CoV-2 sequences were recovered from rRT-PCR positive samples from the cat owners who tested positive for SARS-CoV-2. Therefore, it was not possible to establish a direct link between the virus recovered from the affected animal and the first person diagnosed prior to the animal or second the second person who tested positive for SARS-CoV-2 after the cat became clinical.

## 4. Discussion

Human to animal transmission and natural infections with SARS-CoV-2 have been reported in domestic cats, dogs, ferrets and mink [5,9,27,28], as well as in wild animals in zoological collections around the world including tigers, lions and puma [7,29]. Most natural or experimental SARS-CoV-2 infections in animals, including in domestic cats, have been associated with subclinical- or only with mild clinical signs in affected animals [27,28,29,30,31,32]. Interestingly, among the companion animals with confirmed SARS-CoV-2 infections reported around the world to date, 13 animals died or were euthanized (10 in the US, 2 in Spain and 1 in Brazil) following detection of the virus (including the cat described here) [28,29]. The role of SARS-CoV-2 in the deaths of these animals (eight dogs and six cats) remains largely unknown, however, all animals that tested positive for SARS-CoV-2 and died had underlying clinical conditions or comorbidities, including chronic lung disease, several types of cancers and heart diseases [28,29,33]. These clinical manifestations and outcomes are similar to what has been described in humans with severe COVID-19. Notably, a recent study from England, reporting infections of cats and dogs with the B.1.1.7 lineage of SARS-CoV-2 (alpha variant) identified cardiac abnormalities in the affected animals including congestive heart failure and ventricular arrhythmia [34].

The case described here presents striking similarities (clinical, virological and pathological) with severe cases of COVID-19 in humans [33,35]. Clinically the animal presented severe progressive respiratory distress for 5–6 days and was non-responsive to supportive therapy. Additionally, high viral loads (viral RNA and infectious virus) were detected in upper (nasal and oropharyngeal swabs) and lower (tracheal swab) respiratory secretions and in the lung and heart of the affected cat. In humans, gross lung pathology is usually overlaid by chronic diseases such as chronic bronchitis and emphysema [36] and include heavy, congested and edematous lung, with patchy to diffuse areas of consolidation and occasional arterial thrombosis [36,37,38]. These lesions are similar to the gross findings observed in the lung of the cat in this study; however, no evidence of underlying chronic condition was found in the lung of the animal.

The predominant histologic pulmonary lesion in human patients that develop pneumonia due to the infection with SARS-CoV-2 is diffuse alveolar damage (DAD), including the exudative, proliferative and fibrotic phases, or a mixture of these, at various stages of progression and severity. Common histological changes include edema, congestion and formation of hyaline membranes, hyperplasia of atypical type II pneumocytes (reactive atypia) and increased numbers of macrophages and lesser neutrophils in the alveolar lumen; interstitial thickening with vascular thrombosis, CD4+ and CD8+ lymphocytes and macrophages infiltrates and variable interstitial proliferation of myofibroblasts [36,37,38,39,40]. Similar to the findings in humans, the histological changes observed in the lung of the cat in this study are indicative of DAD, with proliferation of atypical type II pneumocytes (reactive atypia), thrombosis and focal areas of capillaritis and the inflammatory infiltrate being mainly composed of neutrophils and macrophages, which support an early disease stage. An observation that was confirmed here by virus isolation, IHC and ISH, demonstrating active SARS-CoV-2 replication- and suggesting direct viral damage to the lung of the cat.

A characteristic of SARS-CoV-2 infection in humans, is the broad organotropism of the virus which can be detected in the lung and in multiple other vital organs such as heart, liver, brain and kidneys [41]. In the cat reported here, SARS-CoV-2 RNA was detected by rRT-PCR in the lung, heart, kidney, liver, spleen and intestine with high amounts of infectious virus being recovered from lung and heart of the animal. Reported histological findings on the human heart are variable and range from no lesions to a few interstitial mononuclear inflammatory infiltrates [40,42] to myocardial degeneration and necrosis without significant inflammation [39,43,44]. Histological examination of the heart of the cat in this study revealed acute myocardial degeneration and necrosis, with mild interstitial acute inflammation. These observations are compatible with an early manifestation of viral myocarditis but could also be secondary to a disseminated vascular damage. Recovery of high amounts of infectious virus from the heart and visualization of viral particles in the tissue by EM, support viral induced myocarditis. Another interesting finding at necropsy was the presence of pleural effusion (~15 mL of fluid), which is likely a result of the HCM observed in the animal or, conversely, an inflammatory exudate secondary to viral replication in the heart. 

One of the most intriguing findings of this study was the fact that the animal was affected by HCM, as evidenced at necropsy by an enlarged ventricular wall and a narrow left ventricle. The association of underlying cardiovascular disease (CVD) and myocardial injury with severe and often fatal outcomes of COVID-19 in human patients has been well documented [13]. A study exploring the myocardial transcriptomic differences between humans with hypertrophic cardiomyopathy (HCM) and healthy control donors, revealed that ACE2 was the most up-regulated gene in HCM hearts and showed a 5-fold overexpression of ACE2 protein [16]. The authors suggested that increased ACE2 expression in the heart could be associated with an increased risk for severe COVID-19 manifestations in HCM patients [16]. Whether increased levels of ACE2 are expressed in the heart of domestic cats presenting with HCM and whether this condition represents a risk factor to severe disease due to SARS-CoV-2 infection in domestic cats remains to be investigated. However, the fact that HCM has been observed as a comorbidity in other cats diagnosed with SARS-CoV-2 and that presented severe progressive respiratory disease [28,33] support the hypothesis that this common heart condition in cats could potentially predispose severe disease and lead to poor prognosis in domestic cats that become infected with SARS-CoV-2. The clinical, pathological and viral findings on the cat studied here aligns with the algorithm recently developed by CDC [45,46], implicating SARS-CoV-2 with the severe disease outcome that led to humane euthanasia of the animal.

## Data Availability

Data is contained within the article or supplementary material.

## Supplementary Materials

The following are available online at https://www.mdpi.com/article/10.3390/v13081510/s1, Figure S1: In situ hybridization and immunohistochemistry controls. (A) In situ hybridization: left top panel, probe targeting DapB gene from Bacillus subtilis strain SMY was used as a negative control (Advanced Cell Diagnostics catalog no. 310043); right top panel, probe targeting feline host protein peptidylprolyl isomerase B (PPIB) was used as a positive control (Advanced Cell Diagnostics catalog no. 455011); bottom panel, tissue from the SARS-CoV-2 cat probed with the S-gene probe. (B) Immunohistochemestry: left panel, negative cat control lung tissue incubated with SARS-CoV-2 mAb N-specific mAb B6G11; right panel, positive cat control lung tissue incubated with SARS-CoV-2 mAb N-specific mAb B6G11, Table S1: SARS-CoV-2 sequences used for phylogenetic reconstruction and alias used in the phylogenetic tree in Figure S1, Table S2: References and acknowledgements for the SARS-CoV-2 sequences used in phylogenetic analysis.
