# Supplementary material for: Severe SARS-CoV-2 Infection in a Cat with Hypertrophic Cardiomyopathy
Source: Viruses. 2021 Jul 31;13(8):1510. doi: 10.3390/v13081510 (PMC8402861; doi:10.3390/v13081510)
Supplement: Supplementary file 1 [file viruses-13-01510-s001.zip › viruses-1270343-supplementary.pdf]

Table S1.

SARS-CoV-2 sequences used for phylogenetic reconstruction and alias used in the phylogenetic tree in Fig. S1.

Alias	Strain	Alias	Strain
NY-1	USA/NY-MSHSPSP-PV17829/2020	AZ-7	USA/AZ-TG483516/2020
OR-1	USA/OR-OHSU-4261/2020	AZ-8	USA/AZ-TG446877/2020
OR-2	USA/OR-OHSU-4398/2020	AZ-9	USA/AZ-TG448671/2020
OR-3	USA/OR-OHSU-4788/2020	AZ-10	USA/AZ-TG487122/2020
OR-4	USA/OR-OHSU-3793/2020	AZ-11	USA/AZ-TG426533/2020
OR-5	USA/OR-OHSU-4163/2020	AZ-12	USA/AZ-TG493886/2020
OR-6	USA/OR-OHSU-4154/2020	AZ-13	USA/AZ-TG569483/2020
OR-8	USA/OR-OHSU-6566/2020	AZ-14	USA/AZ-TG486581/2020
OR-9	USA/OR-OHSU-5320/2020	AZ-15	USA/AZ-TG493898/2020
OR-10	USA/OR-OHSU-5309/2020	AZ-16	USA/AZ-TG602442/2020
OR-11	USA/OR-OHSU-3965/2020	AZ-17	USA/AZ-TG486899/2020
OR-12	USA/OR-OHSU-4441/2020	IL-1	USA/IL-IDPH-I-000292/2020
AK-1	USA/AK-PHL597/2020	IL-2	USA/IL-IDPH-MAC-S-0001352/2020
WA-2	USA/WA-S3255/2020	DE-2	USA/DE-CDC-2-3713992/2020
WA-4	USA/WA-S4667/2020	DE-3	USA/DE-CDC-2-3713979/2020
WA-5	USA/WA-S4406/2020	SC-1	USA/SC-CDC-2-3714351/2020
WA-6	USA/WA-S5020/2020	MD-1	USA/MD-MDH-0372/2020
WA-7	USA/WA-S3302/2020	ME-1	USA/ME-HETL-J0300/2020
WA-8	USA/WA-S5022/2020	WA-1	USA/WA-UW-23609/2020
WA-9	USA/WA-S4409/2020	WA-3	USA/WA-UW-31214/2020
WA-10	USA/WA-S4127/2020	WA-17	USA/WA-UW-32819/2020
WA-11	USA/WA-S4010/2021	WA-21	USA/WA-UW-60665/2021
WA-12	USA/WA-S4017/2021	NV-1	USA/NV-CDC-LC0005839/2021
WA-13	USA/WA-S4011/2021	VA-7	USA/VA-CDC-LC0006004/2021
WA-14	USA/WA-S3608/2020	WA-18	USA/WA-CDC-LC0013210/2021
WA-15	USA/WA-S4408/2020	WA-22	USA/WA-CDC-LC0013201/2021
WA-16	USA/WA-S4128/2020	DC-1	USA/DC-CDC-LC0000359/2020
WA-19	USA/WA-S4753/2021	OR-7	USA/OR-CDC-2-3693586/2020
WA-20	USA/WA-S4373/2021	IL-3	USA/IL-NM-1708/2020
WA-23	USA/WA-S5046/2020	IL-4	USA/IL-NM-4753/2020
WA-24	USA/WA-S3937/2021	CA-1	USA/CA-ALSR-3879/2020
WA-25	USA/WA-S4046/2021	VA-1	USA/VA-DCLS-1647/2020
UT-1	USA/UT-UPHL-2009714/2020	VA-2	USA/VA-DCLS-1716/2020
UT-2	USA/UT-UPHL-2012051821/2020	VA-3	USA/VA-DCLS-1735/2020
SC-2	USA/SC-DHEC-0007/2020	VA-4	USA/VA-DCLS-1713/2020
DE-1	USA/DE-DHSS-F1006556/2020	VA-5	USA/VA-DCLS-1683/2020
AZ-1	USA/AZ-TG517518/2020	VA-6	USA/VA-DCLS-1744/2020
AZ-2	USA/AZ-TG532109/2020	VA-8	USA/VA-DCLS-1714/2020

AZ-3	USA/AZ-TG532103/2020	VA-9	USA/VA-DCLS-1724/2020
AZ-4	USA/AZ-TG491777/2020	WH01	Wuhan/WH01/2019
AZ-5	USA/AZ-TG403434/2020	Hu-1	Wuhan/Hu-1/2019
AZ-6	USA/AZ-TG490896/2020		

Table S2.
References and acknowledgements for the SARS-CoV-2 sequences used in phylogenetic analysis.

Author	n	publication title	publication URL	strains
Weijun Chen et al	1	Genomic characterisation and epidemiology of 2019 novel coronavirus: implications for virus origins and receptor binding	https://dx.doi.org/10.1016/S0140-6736(20)30251-8	Wuhan/WH01/2019
Zhang et al	1	A new coronavirus associated with human respiratory disease in China	https://dx.doi.org/10.1038/s41586-020-2008-3	Wuhan/Hu-1/2019
Jolene Bowers et al	17	unknown	unknown	USA/AZ-TG517518/2020, USA/AZ-TG532109/2020, USA/AZ-TG532103/2020, USA/AZ-TG491777/2020, USA/AZ-TG403434/2020, USA/AZ-TG490896/2020, USA/AZ-TG483516/2020, USA/AZ-TG446877/2020, USA/AZ-TG448671/2020, USA/AZ-TG487122/2020, USA/AZ-TG426533/2020, USA/AZ-TG493886/2020, USA/AZ-TG569483/2020, USA/AZ-TG486581/2020, USA/AZ-TG493898/2020, USA/AZ-TG602442/2020, USA/AZ-TG486899/2020
SEARCH Alliance San Diego with Tracy Basler et al	1	unknown	unknown	USA/CA-ALSR-3879/2020

Pavitra Roychoudhury et al	4	unknown	unknown	USA/WA-UW-23609/2020, USA/WA-UW-31214/2020, USA/WA-UW-32819/2020, USA/WA-UW-60665/2021
Keith Gagnon et al	2	unknown	unknown	USA/IL-IDPH-I-000292/2020, USA/IL-IDPH-MAC-S-0001352/2020
Erin L. Young et al	2	unknown	unknown	USA/UT-UPHL-2009714/2020, USA/UT-UPHL-2012051821/2020
Chen J et al with Pathogenomics group Dagdag R et al	1	unknown	unknown	USA/AK-PHL597/2020
Ramon Lorenzo-Redondo et al	2	unknown	unknown	USA/IL-NM-1708/2020, USA/IL-NM-4753/2020
Matluk et al	1	unknown	unknown	USA/ME-HETL-J0300/2020
Peter W. Cook et al	5	unknown	unknown	USA/NV-CDC-LC0005839/2021, USA/DC-CDC-LC0000359/2020, USA/VA-CDC-LC0006004/2021, USA/WA-CDC-LC0013210/2021, USA/WA-CDC-LC0013201/2021
Virginia DCLS et al	8	unknown	unknown	USA/VA-DCLS-1647/2020, USA/VA-DCLS-1716/2020, USA/VA-DCLS-1735/2020, USA/VA-DCLS-1713/2020, USA/VA-DCLS-1683/2020, USA/VA-DCLS-1744/2020, USA/VA-DCLS-1714/2020, USA/VA-DCLS-1724/2020
Gregory Hovan et al	1	unknown	unknown	USA/DE-DHSS-F1006556/2020
Krista Queen et al	3	unknown	unknown	USA/SC-CDC-2-3714351/2020, USA/DE-CDC-2-3713992/2020, USA/DE-CDC-2-3713979/2020
Flores et al	1	unknown	unknown	USA/SC-DHEC-0007/2020
Ana S. Gonzalez-Reiche et al	1	unknown	unknown	USA/NY-MSHSPSP-PV17829/2020
Maryland Department of Health Laboratories Administration et al	1	unknown	unknown	USA/MD-MDH-0372/2020

Deborah A. Nickerson et al	19	unknown	unknown	USA/WA-S3255/2020, USA/WA-S4667/2020, USA/WA-S4406/2020, USA/WA-S5020/2020, USA/WA-S3302/2020, USA/WA-S5022/2020, USA/WA-S4409/2020, USA/WA-S4127/2020, USA/WA-S4010/2021, USA/WA-S4017/2021, USA/WA-S4011/2021, USA/WA-S3608/2020, USA/WA-S4408/2020, USA/WA-S4128/2020, USA/WA-S4753/2021, USA/WA-S4373/2021, USA/WA-S5046/2020, USA/WA-S3937/2021, USA/WA-S4046/2021
Brendan L. O'Connell et al	11	unknown	unknown	USA/OR-OHSU-4261/2020, USA/OR-OHSU-4398/2020, USA/OR-OHSU-4788/2020, USA/OR-OHSU-3793/2020, USA/OR-OHSU-4163/2020, USA/OR-OHSU-4154/2020, USA/OR-OHSU-6566/2020, USA/OR-OHSU-5320/2020, USA/OR-OHSU-5309/2020, USA/OR-OHSU-3965/2020, USA/OR-OHSU-4441/2020
Queen et al	1	unknown	unknown	USA/OR-CDC-2-3693586/2020

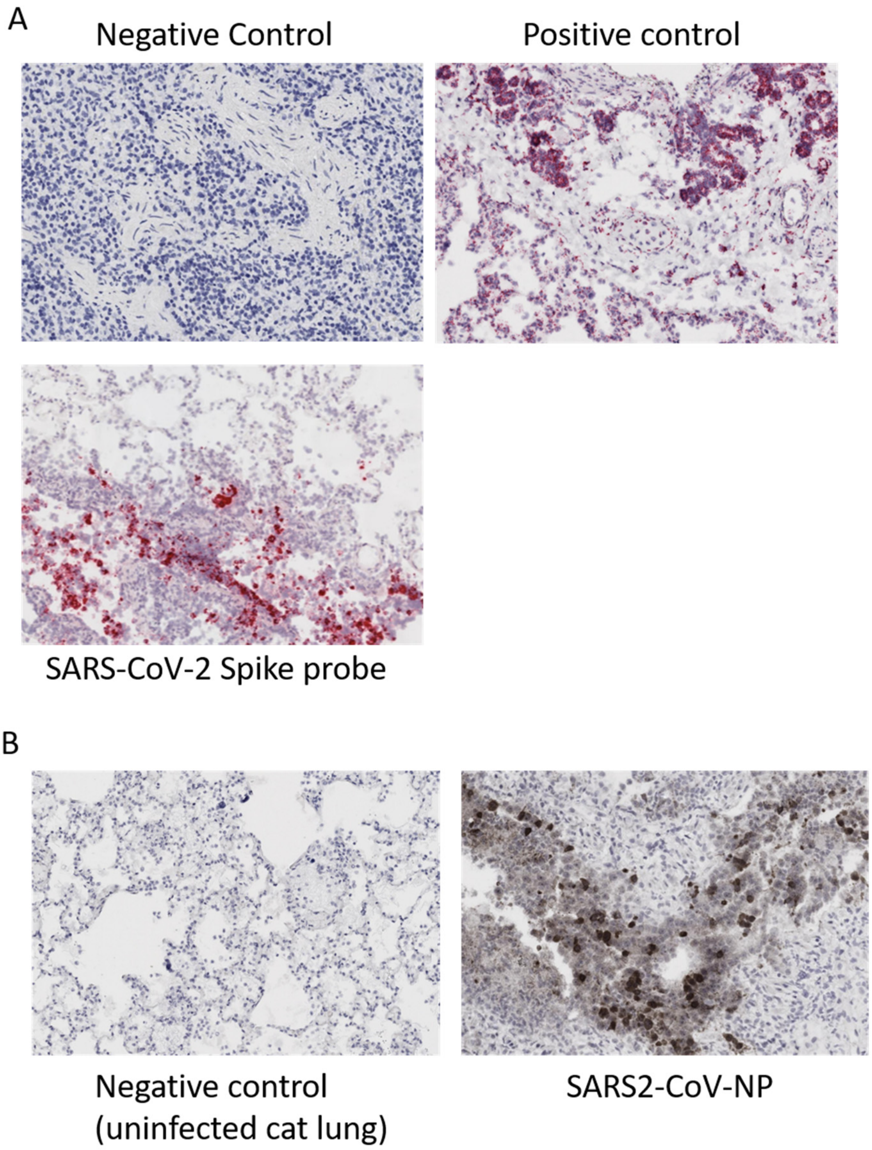

Fig. S1.

In situ hybridization and immunohistochemistry controls. (A) *In situ* hybridization: left top panel, probe targeting DapB gene from Bacillus subtilis strain SMY was used as a negative control (Advanced Cell Diagnostics catalog no. 310043); right top panel, probe targeting feline host protein peptidylprolyl isomerase B (PPIB) was used as a positive control (Advanced Cell Diagnostics catalog no. 455011); bottom panel, tissue from the SARS-CoV-2 cat probed with the S-gene probe. (B) Immunohistochemestry: left panel, negative cat control lung tissue incubated with SARS-CoV-2 mAb N-specific mAb B6G11; right panel, positive cat control lung tissue incubated with SARS-CoV-2 mAb N-specific mAb B6G11.
